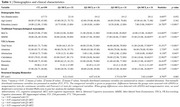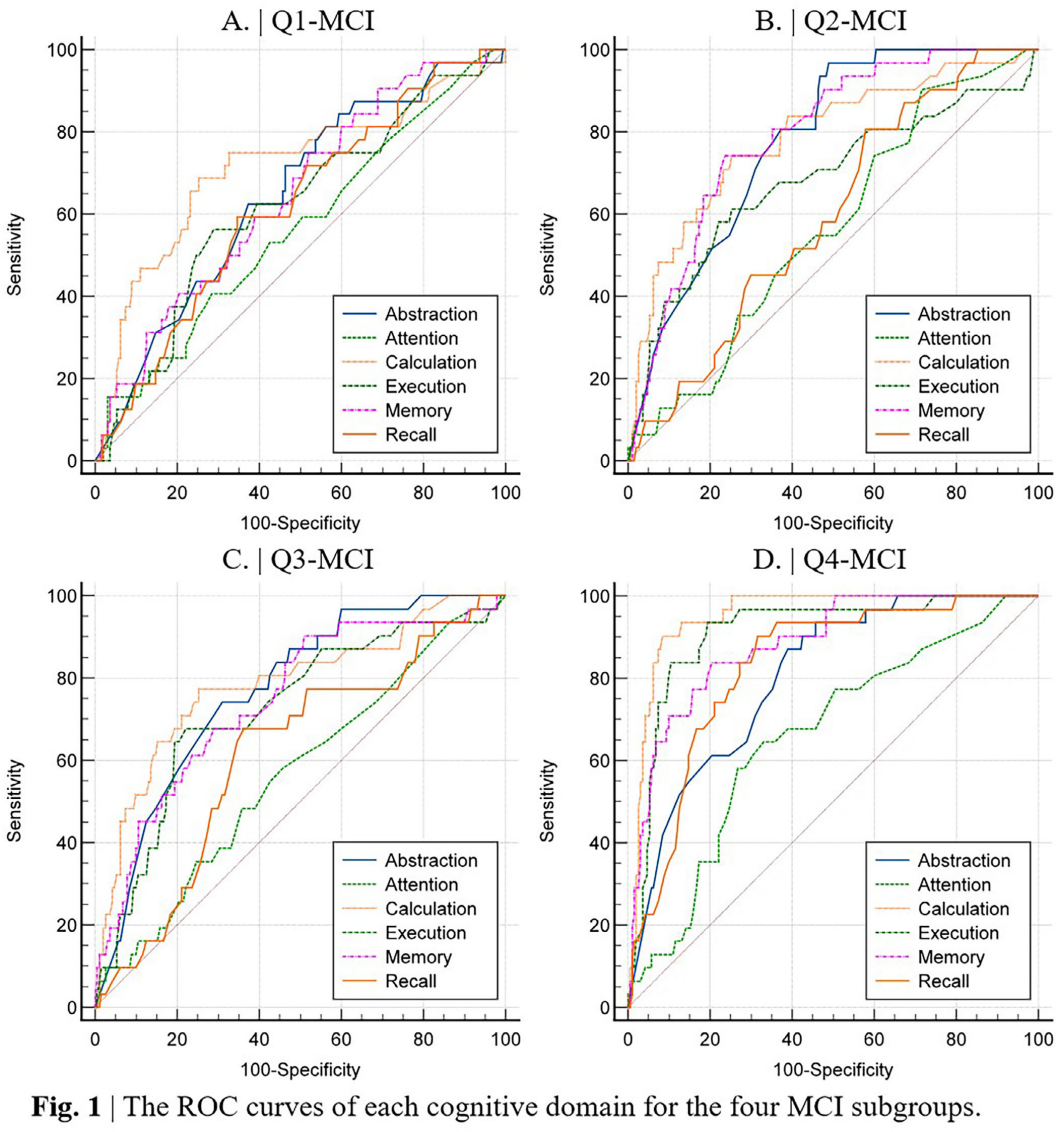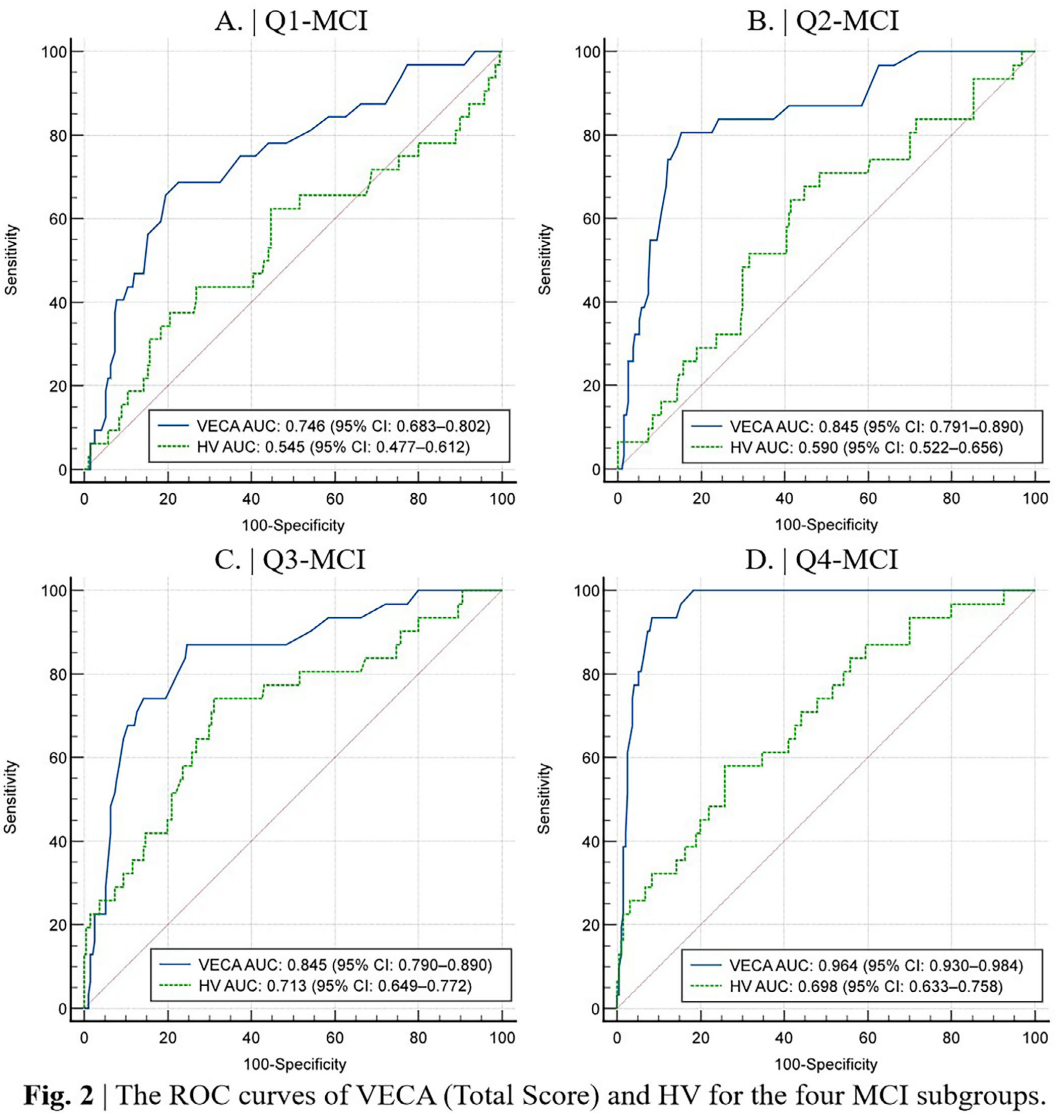# Cognitive Decline Profiles in Mild Cognitive Impairment: A Novel Convenient Multi‐Domain Screening Tool Perspective

**DOI:** 10.1002/alz70856_103384

**Published:** 2025-12-26

**Authors:** Hui Chen, Xiang Fan, Keyan Yu, Zhuonan Wei, Lele Chen, Gaigai Lu, Lin Hu, Tong Wu, Silin Tao, Guanxun Cheng

**Affiliations:** ^1^ Peking University Shenzhen Hospital, Shenzhen, Guangdong, China; ^2^ Shantou University Medical College, Shantou, Guangdong, China

## Abstract

**Background:**

Mild cognitive impairment (MCI) is an intermediate stage between cognitively unimpaired (CU) and dementia, often considered a critical phase for early detection. Mini‐Mental State Examination (MMSE) and Hippocampal volume (HV) are commonly used to evaluate cognitive impairment. However, the sensitivity of these methods for detecting early MCI remains relatively low. Montreal Cognitive Assessment (MoCA) is more sensitive than MMSE in detecting MCI but is also more time‐consuming and complex, requiring greater examiner expertise. There is an unmet need for a screening tool quicker and more convenient than MoCA while being more sensitive than HV. The Virtual Reality Eye‐tracking Cognitive Assessment (VECA) represents a novel approach that integrates virtual reality, eye‐tracking technology, and machine learning to evaluate multiple cognitive domains efficiently. This study aims to explore cognitive decline patterns across MCI subgroups using VECA and compare its diagnostic performance to HV.

**Methods:**

A total of 125 MCI patients and 190 CU individuals from the Shenzhen multi‐modal Aging Research (STAR) cohort underwent neuropsychological assessments, VECA, and 3D‐T1WI MRI. MCI patients were divided into four subgroups (Q1‐MCI to Q4‐MCI) based on their MoCA and MMSE scores. Statistical analyses were performed using SPSS 27.0. The performances were compared using the DeLong test in MedCalc.

**Results:**

In the Q1‐MCI subgroup, functions of abstraction, calculation, execution, memory, and recall consistently performed best. In the Q2‐MCI and Q3‐MCI subgroups, abstraction, calculation, execution, and memory functions consistently performed best. In the Q4‐MCI subgroup, calculation, execution, and memory functions consistently performed best. No significant differences in attention function were observed among the four groups, with AUC values consistently demonstrating lower scores than other cognitive functions (*p* < 0.05). Besides, VECA (Total Score) demonstrated higher AUC values (0.746 for Q1‐MCI subgroup, 0.845‐0.964 for Q2‐MCI to Q4‐MCI subgroup) than HV in all subgroups (*p* < 0.05).

**Conclusion:**

VECA demonstrates strong potential as a rapid and efficient screening tool, requiring only 5 minutes to administer and consistently outperforming HV in differentiating MCI across all stages. VECA findings indicate that attention remains relatively preserved during MCI, while impairments in calculation, execution, and memory become progressively more pronounced in later stages.